# Using sound-taste correspondences to enhance the subjective value of tasting experiences

**DOI:** 10.3389/fpsyg.2015.01309

**Published:** 2015-09-01

**Authors:** Felipe Reinoso Carvalho, Raymond Van Ee, Monika Rychtarikova, Abdellah Touhafi, Kris Steenhaut, Dominique Persoone, Charles Spence

**Affiliations:** ^1^Department of Electronics and Informatics – ETRO, Vrije Universiteit BrusselBrussels, Belgium; ^2^Department of Experimental Psychology, KU LeuvenLeuven, Belgium; ^3^Department of Biophysics, Donders Institute, Radboud UniversityNijmegen, Netherlands; ^4^Philips Research Laboratories, Department of Brain, Body and BehaviorEindhoven, Netherlands; ^5^Laboratory of Acoustics, Division of Soft Matter and Biophysics, KU LeuvenLeuven, Belgium; ^6^The Chocolate LineBruges, Belgium; ^7^Crossmodal Research Laboratory, Department of Experimental Psychology, Oxford UniversityOxford, UK

**Keywords:** taste, sound, music, perception, experience design, gastrophysics

## Abstract

The soundscapes of those places where we eat and drink can influence our perception of taste. Here, we investigated whether contextual sound would enhance the subjective value of a tasting experience. The customers in a chocolate shop were invited to take part in an experiment in which they had to evaluate a chocolate’s taste while listening to an auditory stimulus. Four different conditions were presented in a between-participants design. Envisioning a more ecological approach, a pre-recorded piece of popular music and the shop’s own soundscape were used as the sonic stimuli. The results revealed that not only did the customers report having a significantly better tasting experience when the sounds were presented as part of the food’s identity, but they were also willing to pay significantly more for the experience. The method outlined here paves a new approach to dealing with the design of multisensory tasting experiences, and gastronomic situations.

## Introduction

Chefs and other professionals working in the food industry are increasingly looking to the latest scientific advances in multisensory flavor perception as a source of inspiration for the design of the dining experiences that they deliver to the marketplace (see Spence, 2015b, for a review). A spate of recent studies has highlighted the influence of the sound of the food itself, considering that this can add significant value (not to mention pleasure) to the consumer’s overall multisensory eating/drinking experience (e.g., Spence and Shankar, 2010; Spence et al., 2011; Knight, 2012; Spence, 2015a, for a review).

There is also a growing consensus that the soundscape of the places in which we choose to eat and drink – think, for example, of restaurants and airplanes – significantly affect our perception of taste (Yan and Dando, 2015; see Spence, 2012, 2014; Spence et al., 2014, for reviews).

One of the investigative approaches that have recently been explored is the assessment on how sound modulates taste. This means that, while we eat, the soundscape that we hear may alter, for example, the perceived levels of sweetness and/or bitterness. Crisinel et al. (2012) and Reinoso Carvalho et al. (in press) have shown that it is possible to produce music/soundscapes with the objective of modulating taste perception, using the literature as an underpinning for the sonic stimuli so composed^1^ (i.e., Spence and Shankar, 2010; Knöferle and Spence, 2012). Another study looked for any crossmodal correspondences between classical music and wine (Spence et al., 2013). These researchers demonstrated that pre-existing classical music could be used to modulate the perceived sweetness, acidity, alcohol level, and other elements of a wine.

An additional approach that assesses the influence of sound on taste is based on an analysis of the behavior of consumers. Here, Areni and Kim (1993) have reported that customers are willing to spend significantly more on a bottle of wine when classic music is playing in the background, rather than when ‘Top-40’ pop music is playing instead.

It is important to point out that food presentation is mostly linked with aromas, shapes, colors, and packaging. That being said, recent reports have evaluated whether the value added to food experiences by means of sensory interventions can also be assessed by means of customers’ willingness to pay. For example, Michel et al. (2014) recently concluded that consumers are willing to pay significantly more for art-inspired platting. And such plating techniques are already catching the attention of major retailers, such as Lidl^2^.

The previously mentioned methodologies inspired us to ask new questions, such as: is it possible to add significant value in food products by means of customized soundscapes? And: will participants report that they are willing to pay more for a customized soundscape, while eating?

The research outlined here was designed to assess whether people would, indeed, consider that the music and soundscapes enhanced their tasting experiences. Part of this evaluation assessed, for the first time, whether participants are willing to pay more for a customized soundscape when presented as part of a multisensory tasting experience. In order to clarify the pairing process between sound and food, we also analyzed whether the participants were able to match music, along with food, in specific geographical, and cultural contexts. Additionally, we considered whether the customers’ cultural background and their prior knowledge about the food that was being tested, might influence the consumer’s response to a multisensory tasting experience. Envisioning a more ecological approach, the present experiment was performed in a chocolate shop with the store’s own soundscape as one of the auditory stimuli. Moreover, we analyzed the feasibility of predicting how a pre-existing song – produced in popular formats without any obvious relation to food and drink – may be used to modulate taste.

## Materials and Methods

### Participants

The experiment, which was conducted at The Chocolate Line Shop (Meir 50, 2000 Antwerp, Belgium), was approved by the Social and Societal Ethics Committee at KU Leuven (SMEC). On the 5th and 6th May, 2015, the customers who visited the store were invited to take part in a short experiment. They were informed that they would be given complimentary chocolates to taste while sometimes listening to sound, and answering a survey. Two hundred and nineteen participants (62% females, mean age of 30 years, *SD*: 13.2 – all of the participants were at least 16 years of age) gave their informed consent prior to taking part in the study. None of the customers who had previously consented to take part in the experiment disagreed with the informed consent. None of the participants reported having a cold or any other impairment of their senses of smell, taste, or hearing at the time of the study. Based on their language skills^3^, most interviewed clients were regional tourists from Belgium, The Netherlands, and Germany, a profile that matches the typical customer base of this shop. Moreover, 80.4% of the customers did not know the shop’s owner – the award-wining chocolatier Dominique Persoone (www.thechocolateline.be) – and, on a 7-point scale that assessed how often they consumed products from the shop, 78% rated 1 or 2 (1 denoting ‘never’), meaning that most of the participants had not experienced products from “The Chocolate Line” previously (Mean: 2 *SD*: 1.6).

### Stimuli

The selection of the food sample was based on a discussion between the first author and the chocolatier, who agreed to produce the taste stimuli for this experience. This discussion focused on ways of pairing sound and chocolate taste and, somehow, linking this matching process with the identity of the chocolatier and his brand, ‘The Chocolate Line.’ The discussion covered aspects of the chocolatier’s professional life that could be seen as having an emotional connection to the creative process involved when he conceives his chocolate formulas. After several attempts involving tasting different types of chocolates, producing, selecting, and listening to different songs and soundscapes, it was decided to focus on the fact that Dominique is known as an experimental chocolatier, and also as an adventurer (he presents a TV show where he travels around the world and explores different cultures, constantly looking for the ‘ultimate cacao bean’). Given that The Chocolate Line imports a majority of its cacao from their Latin American plantation, it was decided that a suitable match should also resemble tropical conditions and elements of Latin American culture.

#### Taste Stimuli

**FIGURE 1:**
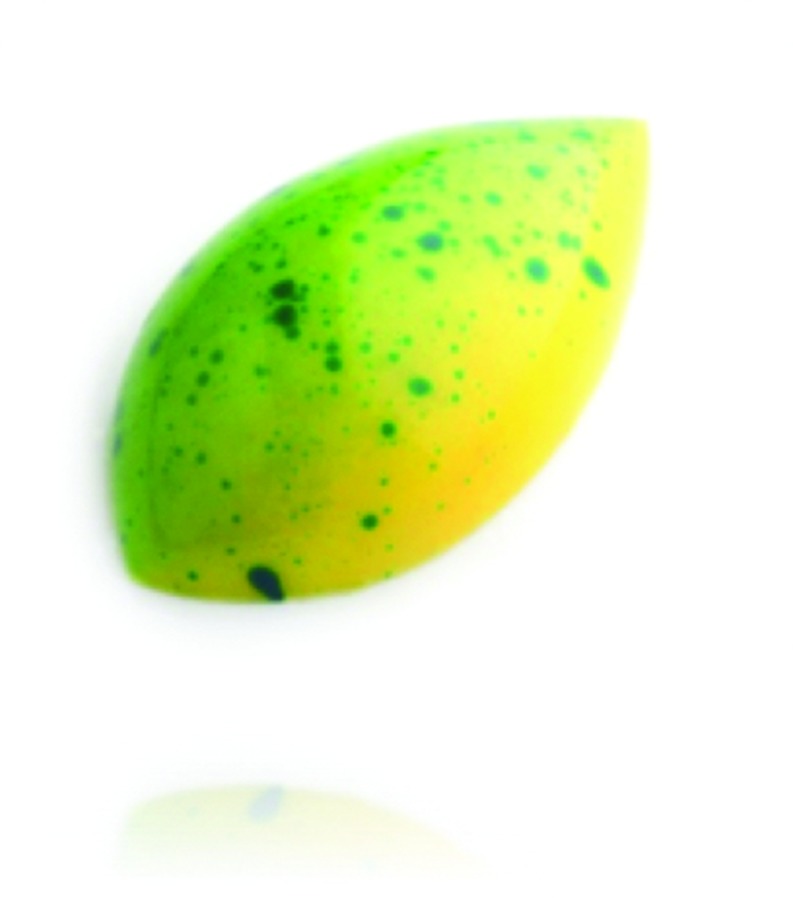
**Visual aspect of the chocolate sample that was used in the experiment**. Here, it is possible to visualize the external color grade-scale and an overall rounded shape, with pointed tips.

The chosen chocolate sample was based on an existing Belgian praline that is part of the special collection of the aforementioned chocolatier – the reference praline is named ‘Brasil’^4^. This praline consists of a colored mass based on white chocolate, together with a mixed filling of lime jelly and coriander ‘ganache’. Its external appearance is composed of green, yellow, and blue speckles. The praline can be seen in **Figure 1**. The formula is provided in **Table 1**.

**Table 1:** Description of chocolate praline ingredients, based on the original recipe.

	Ingredient	Amount (% of mix)
Lime jelly	Lime juice	50
	Jelly sugar	50
Coriander ganache	Cream	27.5
	Coriander	3.7
	Glucose	4.7
	White chocolate	56.5
	Crémoline	2
	Espelette pepper	0.8
	Cachaça	3.9
	Coriander alcohol	0.8
Color mass	Cocoa butter	50
	White chocolate	50
	Blue dye (natural)	-
	Yellow dye	
	Green dye	

*For the molding, a mix of one part of white chocolate with one part of cocoa butter was used. The formed mass was sprayed – one side in yellow and the other side in green – and blue speckles were then added. For the filling, the lime juice was boiled and then jelly sugar added. Once the jelly was cold, one third of the mold was filled with it. For the rest of the filling, the cream and the glucose were boiled and the coriander was added. The chocolate, crémoline, and espelette pepper were then poured in. After mixing well, the coriander alcohol was added to the cream. The form was filled up to 1mm of the edge and allowed to set for a day. Finally, the form is closed with white chocolate before it was taken out of the form*.

#### Auditory Stimuli

**FIGURE 2:**
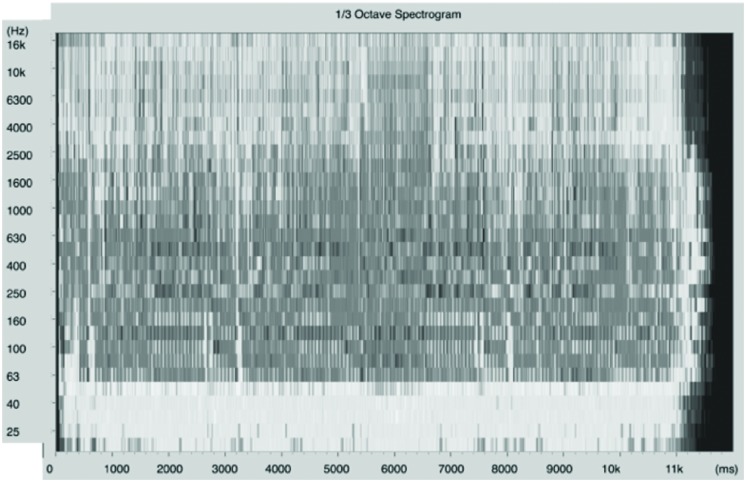
**Spectral and temporal features of the song**. Co-relating the psychoacoustic analysis with the summary of the crossmodal correspondences between basic tastes and sonic elements presented by Knöferle and Spence (2012), the suggestion would be that the song might enhance the perceived sweetness of the chocolate. Due its syncopated rhythm and relatively fast tempo, the song may also enhance the perceived sourness (Figure source: dBSONIC).

A fragment of “Vem Morena, Vem,” a song composed by the Brazilian artist Jorge Ben4, was chosen for this experiment. The song appears on the album “Samba Esquema Novo” released in 1963, produced by Armando Pittigliani (Philips, Universal, re-mastered and re-released by Polysom in 2012). The song (and indeed the entire album) is an example of bossanova rhythms and choruses blended together. It comes along with a big-band-ensemble and the artist’s signature minor-tone arrangements. The lyrics refer to a musician who is calling the attention of a lady, inviting her to dance ‘samba,’ while at the same time exalting his/her own musical skills. The lyrics are presented in two main stanzas that are developed throughout the song^5^. As Travis Drageset describes in his overview at Allmusic^6^, the album “propels the set into an upbeat and enjoyable listen.” For its precise psychoacoustic characterization, the psychoacoustic model provided by the dBSONIC package was used (manufactured by 01 dB Metravib). From this analysis, it was possible to extract the following parameters. The average levels of the song’s sound pressure fluctuated between 64 and 72 dBA 78% of the time (68 ± 4 dBA). The loudness curve fluctuated between 13 and 26 phones during 84% of the time (the referred phone values correspond to the ear response curves for low sound pressure levels, levels commonly associated to a pleasant hearing). Sharpness is 1 Acum during 90% of the track (low sharpness). Finally, roughness and fluctuation strength were distributed homogeneously throughout the song, from which it can be assumed that there shouldn’t be either rapid or slow significant transients provoking subjective perception of amplitude modulation (meaning balanced-low roughness). **Figure 2** shows the spectral and temporal features of the song.

### Procedure

**FIGURE 3:**
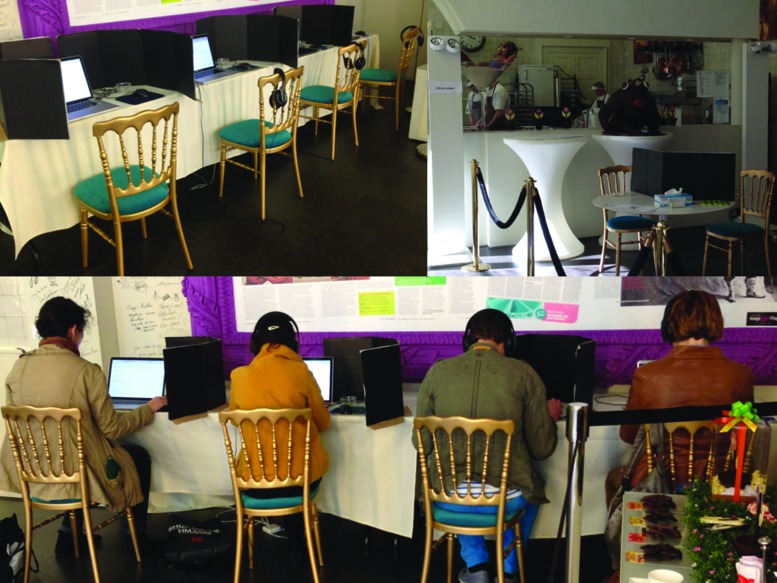
**Experimental booths prior to the experiment **(top left)**, overview of the kitchen when entering the experimental area **(top right)** and overview of the experimental booths during the experiment (bottom)**.

The Chocolate Line Shop Antwerp is subdivided in three main rooms. For our set up, the back room was chosen. This room has a circulation area for guided visits and a production kitchen, where different types of chocolate are prepared on a daily basis. Visitors can visually appreciate the work that takes place in the kitchen through a glass partition. The room’s soundscape is mostly composed of the sounds of the working kitchen. Four experimental booths were placed in the circulation area, where four participants entered at once. The booths were separated in order to prevent communication between the participants. While the experiment was being conducted, access to the mentioned area was restricted to the kitchen workers, participants, and supervisors. The intensity of the artificial lighting in the experimental area was dimmed in order to provide a more ‘intimate’ ambience. **Figure 3** shows the configuration of the experimental booths and an overview of the production kitchen.

In each booth, the participant was seated in front of a computer screen. Each participant had a pair of headphones, a computer mouse, and a keyboard to interact with the survey. Each sound reproduction system was calibrated by a CESVA SC310 sonometer to ensure that each participant was exposed to the soundtracks at the same sound pressure level (*L*_At2min_ = 73 ± 3 dBA). The soundtracks were presented over Sennheiser HD 202 headphones. The survey consisted of an electronic form containing three main steps, and 7-point Likert-scales/YES-NO questions. In the first step, the participants had to input their personal information, read and accept the conditions of the informed consent. In a second step, they had to respond to a pre-questionnaire, in which they evaluated the visual aspects of the chocolate sample prior to eating it. Part of this visual evaluation included reporting how much they were willing to pay for the chocolate. As a reference, it was indicated to them that the average price of a chocolate praline at this shop was 0.66€ per unit. In this pre-questionnaire, they also had to describe their customer profile (e.g., how often they bought products from this chocolate shop, etc. – see supplementary material for detailed questionnaire). During these two first steps, all of the participants were exposed to the same conditions. The third step of the experiment consisted of a between-groups design, in which the participants had to respond to questions while eating the chocolate in each of the four experimental conditions. In the first condition (A), the participants tasted the chocolate while listening to the song. No extra information about the stimuli was presented. The second condition (B) involved tasting the chocolate while listening to the ambient soundscape of the production kitchen. The participants in the third condition (C) ate the chocolate while listening to the song. However, this time they were told that the song had actually been the chocolatier’s source of inspiration when creating the chocolate sample that they were about to taste. For the fourth condition (D), the participants ate the chocolate while listening to the song, and they were told that the song had been chosen by a team of scientists because of its enhancing effect on the taste of the chocolate.

During the development of this method, several factors that could have had an impact on the concentration levels of the participants were considered. For instance, the experiment was to be performed inside a busy chocolate shop, during working hours. The room to be used had natural light and visual decorative details that could not be altered. Finally, the assessment of how sound can influence taste does not necessarily come as an easy association to naive participants, from which we had to find ways to focus their attention on the expected multisensory effects. Therefore, in order to highlight the insertion of a customized soundscape into the creative process of the chocolate sample, along with the multisensory protocol described in the paragraph above, in conditions B–D the participants were also told that the chocolate they were about to eat had been hand-crafted by the chocolatier.

Two supervisors were present during the entire process to provide extra guidance, coordination, and support, together with the written guidelines concerning the experiment.

As a final question, the participants were asked whether they desired another chocolate sample. Upon finishing the experiment, they were instructed to leave the room without discussing any details with the next group of participants. Paper towels for hand cleaning were available before and after the experimental procedure.

## Results

Before eating, the participants rated the chocolate visually. After having the multisensory experience, the participants again rated how much they liked the chocolate (one being ‘not at all’ and seven being ‘very much’). On average, the participants rated liking the chocolate significantly more after tasting it (*p* < 0.05). **Table 2** shows the means and *SD* of these comparisons, per condition^7^.

**Table 2:** Comparison of rates (Means and *SD*) before and after tasting the chocolate.

	Condition			
	A	B	C	D
Pre-tasting	3.8 (1.5)	4.6 (1.6)	4 (1.5)	3.9 (1.4)
Post-tasting	**4.9 (1.5)**	**5.3 (1.6)**	**5.4 (1.3)**	**4.9 (1.4)**

*Values in bold were reported after the participants had tasted the chocolate. If Comparing the answer to the pre-questionnaire ‘tastiness’ item with their ‘liking’ while eating, revealed statistically significant results for each condition, when considered individually (*p* < 0.05). The statistical analysis is based on a comparison of means between the related groups (paired samples t-test, using SPSS)*.

The participants would have been willing to pay an average of 0.59€ in the pre-questionnaire (*SD*: 0.16). The average price after having had the multisensory experience was 0.65€ (∼10% higher, *SD*: 0.20), and in condition C it was 0.71€. The price difference was significant (*p* < 0.05). **Table 3** shows the means and *SDs* for each condition. Note that before eating, the participants rated how much they were willing to pay for the chocolate. After eating, they were asked to rate the ‘chocolate experience,’ making it clear that the second answer should consider the sonic stimulus involved.

**Table 3:** In conditions A and B, the participants were willing to pay approximately 10% more for the chocolate when listening to customized sonic stimuli.

Condition	Pre-tasting	A	B	C	D
Mean (*SD*)	0.59 (0.16)	0.65(0.20)	0.65 (0.20)	0.71 (0.20)	0.61 (0.20)

*In condition C, they were willing to pay almost 20% more for such an experience. A comparison of means was obtained to assess the difference of prices between the willingness to pay prior tasting and conditions A–D, individually. Significant differences were reported in all cases for p < 0.05 (p_PA_ = 0.014, p_PB_ = 0.018, p_PC_ = 0.001, p_PD_ = 0.002)*.

When eating, the participants matched the taste of the chocolate using two different categories. The first category was related to country (i.e., China, Morocco, Brazil, etc.) and the second was related to a particular geographical context (i.e., tropical, urban, beach, etc.). **Table 4** shows that there was a stable trend when matching countries. However, no such trend was apparent when relating the chocolate experience with a geographical condition.

**Table 4:** When matching the chocolate to a country, between 72 and 79% of the participants chose Brazil.

	Country (in %)		Geographical context (in %)	
	Brazil	Other	Tropical	Other
A	77	23	54	46
B	72	28	79	21
C	79	21	65	35
D	75	25	70	30

*Yet, when matching this taste with a geographical context, the range of choices increased considerably amongst the various conditions, from 54 to 79%. Furthermore, in conditions A,C, and D, an average of 5% more participants matched the chocolate’s taste with Brazil, as compared with condition B. That being said, we could presume that the song may have helped to narrow their choices, thus giving rise to more accurate matches*.

In conditions A, C, and D, the participants answered questions regarding their eating experience while listening to the song. Ninety-five percent of the participants declared that they were unfamiliar with the song. However, most of them agreed that it matched the taste of the chocolate (81% rated equal or more than 4 points, with 7 indicating ‘very much’; Mean: 4.8 *SD*: 1.6). When asked to associate the song with different geographical regions (i.e., Northern/southern Europe, Africa, North/Latin American, etc.), an average of 73% associated the song with Latin America. When asked if they liked the song, an average of 81% rated equal or more than 4 points (7 being ‘very much’; Mean: 4.7 *SD*: 1.6). And, when asked how much they were generally interested in music, 85% of the participants rated equal or more than 4 points (7 being ‘very much’; Mean 5 *SD*: 1.4). **Table 5** shows these ratings for all conditions.

**Table 5:** The participants in Condition C were the one’s who reported being most familiar with the song, and most interested in music generally.

Condition	A	C	D
Do you know this song?			
% No	98	**90**	96
How much are you interested in music?			
Mean	4.7	**5.25**	4.9
How much do you think this song matches the chocolate’s taste?			
Mean	4.8	**4.9**	4.7
Match the song with one of the options:			
% Latin America	68	**77**	71
How much do you like this song?			
Mean	4.5	4.8	4.8

*The participants in condition C were also the ones with the highest ratings when matching the song with the chocolate’s taste, and the ones that matched Latin America as the song’s most suitable context. Therefore, a stronger connection between the participants and the sonic stimuli may further be expected to improve the matching process. These results suggest that the participants in Condition C were the ones who were most familiar with the song and that they were also the ones performing a more accurate matching*.

Prior studies have demonstrated that taste ratings can be influenced by means of customized sonic cues, that had been produced for such purposes (Crisinel and Spence, 2009, 2010, 2012; Bronner et al., 2012; Crisinel et al., 2012; Reinoso Carvalho et al., in press). Here, we assessed the effects on taste of a song that had not been produced explicitly to modulate taste. The participants rated the basic flavor components of the chocolate while tasting it (Mean sweetness: 4.55 *SD* 1.5, Mean bitterness: 2.3 *SD* 1.3, Mean sourness: 4.1 *SD* 1.5, and Mean saltiness: 2.3 *SD* 1.3; with 1 corresponding to ‘not at all’ and 7 to ‘very much’). When comparing the modulatory effects of the song between conditions, reports concerning perceived sweetness are worth mentioning. The participants who heard the kitchen soundscape (condition B) instead of the song (conditions A, C, and D) rated the chocolate as tasting less sweet (Mean B: 4.33, *SD*: 1.4, versus Mean ACD: 4.62, *SD*: 1.5), although this comparison failed to reach statistical significance. The comparisons between the ratings on the levels of bitterness, sourness, and saltiness, among conditions, were inconclusive. Nevertheless, a remarkably high level of sourness was reported (on average, the levels of sourness were rated almost as high as the sweetness, in all conditions). Along with the chocolate’s formula (see **Table 1**), it is feasible that part of the perceived sourness may be associated with the green and yellow colors of the chocolate’s presentation, along with the pointed tips of the praline’s design (see Spence and Ngo, 2012; Spence et al., 2015, for an overview on how colors and shapes can influence the perception of taste, respectively). That being said, due to general expectations concerning how a chocolate should taste, it is possible that the overwhelming perceived sourness may have demanded extra attention from the participants, thus potentially compromising the modulatory effect of the song on the levels of sweetness of the chocolate’s taste. As recently noted by Spence (2015a), our flavor expectations can have a profound influence on our daily taste/flavor experiences (see Stevenson, 2009; Piqueras-Fiszman and Spence, 2015, for reviews).

Analysis of the results suggested that condition C may have had a greater impact on the overall multisensory experience. The participants in Condition *C* not only gave the highest liking ratings after tasting, but the differences between their ratings prior to and after tasting were also the highest. They were also the ones with the highest ratings when matching the song with the chocolate’s taste. Furthermore, they were the participants with the most accurate matching: 79% of the participants in Condition C matched the chocolate with Brazil and 77% matched the song with Latin America. From this, it can be assumed that they were the ones that had the most gratifying experience. In summary, those participants who had been told that the song was used as the source of inspiration by the chef (condition C), reported liking the chocolate the most after eating it, and were also the ones who were willing to pay the most for this experience.

## Discussion

The results of the present study highlight a clear distinction between the ratings of the participants before eating the chocolate and after having enjoyed the multisensory tasting experience. When stimulated by a song and a soundscape that were part of the identity of the chocolate, and when told that the chef himself had hand-crafted the food, not only did they experience sound as part of a context, but they also had the opportunity to learn – and, somehow, to share – the creative process involved during the development of the product that they had tasted. Under such conditions, sound can be considered as a sensory link between the chef’s creative process and his (or her) customers’ tasting experience.

It is also clear from the present results that the expected association between the chocolate and the song was well received by the majority of the participants. Even though most of them reported being unfamiliar with the chocolate, the chocolatier, or the song, they were nevertheless able to clearly distinguish the expected cultural and geographical context of both, in all conditions.

Most of the participants reported being genuinely interested in music, although the majority of them had no direct cultural connection with the referred context, and most of them also reporting being unfamiliar with the song that was presented here. These rates contrast with their reports concerning their knowledge of chocolate^8^. Therefore, customers may feel genuinely confident in participating in those experiences that involve food and music, regardless of their actual musical expertise. These results also imply that a stronger familiarity with the song may have resulted in them feeling even more confident (see **Table 5**). The experimental approach outlined here will hopefully inspire more chefs and food industry professionals to consider the further adoption of customized music in food experiences. Music could be used as an element that adds sensory value to a product without the need to alter its visual and/or aromatic appearance.

As mentioned previously, we believe that more participatory approaches, such as those that consider environmental aspects of everyday experiences, may also encourage the development of similar experiences in actual gastronomic situations (Reinoso Carvalho et al., 2013, 2015, in press). Benefitting from a pre-existing soundscape that is part of the shop’s scenario – as in the case of the multisensory tasting experience reported here – may trigger new ways of dealing with sensory branding. Here, we can additionally mention the importance of using popular music in behavioral tests. In previous research, it has been shown that it is possible to produce music that can have a modulatory effect on taste perception. But, in order to make these experiences more inclusive for the general public, it is crucial to focus on developing research with existing popular music, since people spend most of their time listening and consuming music that was not necessarily produced with gastrophysics in mind.

Some of the previous studies that we considered as source of inspiration faced similar challenges in interpretation as we did. We found that the song may have had a modulating effect on the levels of sweetness of the chocolate’s taste, although this comparison failed to reach statistical significance. Crisinel et al. (2012) reported significant differences in the levels of sweetness and bitterness of toffee, but did not report significant differences when asking to the participants if the position of the flavor changed inside their mouth when switching the auditory stimulus, and neither when comparing how much the participants liked the flavor of the food while listening to the two different soundtracks. Reinoso Carvalho et al. (in press) reported significant differences in the modulation of taste in results corresponding with the most bitter chocolate sample, when three different chocolate formulas were being tested. Meanwhile, the second experiment reported by Spence et al. (2013), did not find significant differences between the ratings with or without music for all of the assessed attributes of the three wines being tested, such as the sweetness, acidity and alcohol levels of the ‘Domain Ponsot, Clos de la Roche 2009.’ Therefore, we believe that, for a better understanding on how the senses of taste and audition interact, further research in this area is still needed. Moreover, in our case we have chosen to include, along with the multisensory experience, the message that the chocolate was being hand-crafted by the chocolatier. Even though the majority of the participants reported not knowing him and never having tasted one of his products, such message may have had an effect on the participant’s behavior. Future research could also tackle the impact of disentangling both factors.

Finally, the fact that consumers are willing to pay significantly more for food accompanied by customized soundtracks, not only opens new doors for scientific analysis and assessment of the sensorial meaning of such results, but also opens opportunities for rethinking the consumption of music through multisensory media. In our case, when comparing the results of condition A with conditions B, C, and D, we found that not only were people willing to pay up to 20% more for a chocolate that comes with its own sonic identity, but they also reported having a significantly better tasting experience.

## Conclusion

The study reported here examined the role of popular music and contextual soundscapes in the subjective ratings of multisensory tasting experiences. The results demonstrate that the participants respond positively when sonic stimuli were presented as part of the identity of a food product. Here, we also show that consumers are willing to pay significantly more for food experiences accompanied by customized and contextualized soundscapes. This method may inspire new ways of dealing with the future design of multisensory experiences, user-studies in gastrophysics, and possibly also actual gastronomic situations.

## Conflict of Interest Statement

The authors declare that the research was conducted in the absence of any commercial or financial relationships that could be construed as a potential conflict of interest.

## Footnotes

1 In the study presented by Reinoso Carvalho et al. (in press), three soundtracks were produced, one designed to be congruent with sweetness, another with bitterness, and the third somewhere in-between.

2 Retrieved from http://www.dailymail.co.uk/news/article-3168913/If-meal-looks-good-think-tastes-better-Simply-arranging-food-carefully-plate-persuade-diners-pay-three-times-it.html (July, 2015).

3 95% of the participants answered the survey in English or Dutch (Spanish, Portuguese and French versions were also available), from which 64% declared Dutch as their mother tongue. Moreover, 75% declared their fluency in English, 22% in French, and 19% in German.

4 The name of the chocolate sample, the title and origins of the used song were not disclosed during the course of the experiment, in order to avoid bias.

5 Retrieved from http://letras.com/jorge-ben-jor/86455/ (July, 2015).

6 Retrieved from http://www.allmusic.com/album/samba-esquema-novo-mw0000513052 (May, 2015).

7 In **Tables 2**–**5**, the values associated to A–D correspond to the results of the four experimental conditions, that are the outcomes of the between group’s part of the experiment.

8 The participants were asked to rate how much of an expert they considered themselves in terms of types and brands of chocolate. On a 7-point scale (1 being ‘Not at all’), the mean of ratings was 2.7 (*SD* = 1.5).
