# Supplementary material for: Using sound-taste correspondences to enhance the subjective value of tasting experiences
Source: Front Psychol. 2015 Sep 1;6:1309. doi: 10.3389/fpsyg.2015.01309 (PMC4554938; doi:10.3389/fpsyg.2015.01309)
Supplement: Supplementary file 1 [file Data_Sheet_1.PDF]

Supplementary Material

Using Sound-Taste Correspondences to Enhance the Subjective Value of Tasting Experiences

Felipe Reinoso Carvalho, Raymond Van Ee, Monika Rychtarikova, Abdellah Touhafi, Kris Steenhaut, Dominique Persoone, & Charles Spence*

*** Correspondence**: Felipe Reinoso Carvalho **freinoso@vub.ac.be**

1. Questionnaires

1.1 Questionnaire prior eating

How much do you like the presentation of the chocolate?
(7 - Very Much / 1 - Not at all)

How tasty does this chocolate look?
(7 - Very Much / 1 - Not at all)

Do you know the chocolatier Dominique Persoone?
YES/NO

How often do you consume products from The Chocolate Line?
(7 - Very Often / 1 - Never)

How much would you be willing to pay for this chocolate?
(At The Chocolate Line Shop, the average price of the products is 0.66€ per unit)

9. () 0.4€
8.() 0.5€
7.() 0.55€
6.() 0.6€
5.() 0.65€
4.() 0.7€
3.() 0.8€
2.() 0.9€
1.() 1€
Other ( )

1.2 Questionnaire after eating

How much do you like this chocolate experience?
(7 - Very Much / 1 - Not at all)

How sweet was the chocolate’s taste?
(7 - Very Much / 1 - Not at all)

How salty was the chocolate’s taste?
(7 - Very Much / 1 - Not at all)

How bitter was the chocolate’s taste?
(7 - Very Much / 1 - Not at all)

How sour was the chocolate’s taste?
(7 - Very Much / 1 - Not at all)

Do you know this song?$^{+}$
YES/NO

How much do you like this song?$^{+}$
(7 - Very Much / 1 - Not at all)

How much do you think this song matches with this flavor?$^{+}$
(7 - Very Much / 1 - Not at all)

Please, match the song with one of the following options:
North America ( )
Latin America ( )
Africa ( )
Australia ()
Asia ()
Southern Europe ( )
Northern Europe ( )

Please, match the chocolate with one of the following options:
Tropical ( )
Urban ( )
Beach ( )
Desert ()
Snow ()
Other ()

Please, match the chocolate with one of the following options:
Brazil ()
China ( )
Morocco ( )
Germany ( )
Australia ()
Other ()

How much of an “expert" are you in chocolate types and brands?
(7 - Very Much / 1 - Not at all)

How interested are you in music?[+]
(7 - Very Much / 1 - Not at all)

How much would you be willing to pay for this chocolate? (price per unit)
9. () 0.4€
8.() 0.5€
7.() 0.55€
6.() 0.6€
5.() 0.65€
4.() 0.7€
3.() 0.8€
2.() 0.9€
1.() 1€
Other ( )

Do you want another piece of the same chocolate?
YES / NO
(In case your answer is YES, before leaving just ask the person in charge for another sample)

The questions marked with the symbol ‘[+]’, were not part of the questionnaire correspondent to condition B.
